# Development and Validation of Species-Specific KASP and SCAR Markers for the Rapid Identification of the Endangered Orchid *Calanthe aristulifera*

**DOI:** 10.3390/plants15101453

**Published:** 2026-05-10

**Authors:** Jung Eun Hwang, Seongjun Kim, Hyeong Bin Park, Sung Min Han, Yubin Lee, Chang Woo Lee, Young-Joong Kim

**Affiliations:** 1 Research Center for Endangered Species, National Institute of Ecology, Yeongyang 36531, Republic of Korea; 2 Department of Environmental Horticulture, University of Seoul, Seoul 02504, Republic of Korea; 3 Department of Native Plant Ecology, National Institute of Ecology, Seocheon 33657, Republic of Korea

**Keywords:** allele-specific PCR, conservation genetics, cryptic hybridization, genotyping-by-sequencing (GBS), sequence-characterized amplified region (SCAR)

## Abstract

*Calanthe aristulifera* is a critically endangered orchid species of profound horticultural and ecological significance. However, establishing its species integrity is complicated by frequent natural introgression with sympatric relatives, such as *C. sieboldii* and *C. discolor*. Because phenotypic plasticity and complex hybrid swarms often confound traditional floral phenotyping, establishing an accurate molecular diagnostic system is imperative for conservation. In this study, we developed and validated high-throughput Kompetitive Allele-Specific PCR (KASP) and rapid Sequence-Characterized Amplified Region (SCAR) markers utilizing Genotyping-by-Sequencing (GBS) data from 64 *Calanthe* individuals—the same dataset reported in a companion population genomic study—re-analyzed using a more stringent marker-development pipeline. From 853,301 SNPs and 55,857 InDels initially identified, we filtered 62,231 high-quality SNPs and 1271 InDels to mine fixed homozygous alleles specific to *C. aristulifera*. This process isolated 179 SNP and 107 InDel loci to design three KASP markers (Ca-KASP1–3) and two SCAR markers (Ca-SCAR1–2). The KASP assays demonstrated a concordance of 98.4% (63/64 individuals; 95% CI: 91.6–99.7%) with morphological pre-classification. The single discordant case (Sample 52) was independently confirmed as a heterozygous hybrid by GBS-based population genomic analysis of the same individuals, providing molecular ground truth entirely independent of morphological assessment. The combined SCAR marker system yielded 96.9% concordance (62/64; 95% CI: 89.3–99.1%). Our findings provide an essential molecular framework for assessing species integrity and guiding the restoration of endangered *C. aristulifera* populations.

## 1. Introduction

Orchids belonging to the genus *Calanthe* exhibit exceptional horticultural, medicinal, and ecological significance globally [1,2]. Among them, *Calanthe aristulifera* is a terrestrial evergreen orchid with a highly restricted distribution in the warm temperate evergreen forests of Korea, Japan, and China, and it is currently facing a severe threat of extinction [3]. Morphologically, this species is characterized by a unique downward-pointing spur and demonstrates an evolutionary reproductive strategy of sequential flowering from the bottom of the inflorescence upward, which prevents self-pollination and promotes cross-pollination. However, natural populations have drastically declined due to over-collection for horticultural value and habitat destruction. Furthermore, recent long-term monitoring studies indicate that the extreme scarcity of pollinators in its native island habitats results in an exceedingly low natural fruit set, posing a critical threat that hinders natural population regeneration [4]. Consequently, this species is designated as a strictly protected endangered species.

The challenge of conservation is further compounded by the difficulty of accurate species delimitation. From a cytological perspective, *C. aristulifera*, *C. sieboldii*, and their natural hybrid *C.* × *kibanakirishima* are all diploids with 2n = 40 chromosomes [3,4], meaning that ploidy-level differentiation cannot account for the morphological variation observed among these taxa. Instead, the intermediacy of *C.* × *kibanakirishima* reflects interspecific gene flow, which renders morphology-based identification unreliable without the support of molecular tools. To establish successful species restoration and systematic conservation strategies, it is urgently required to accurately elucidate the genetic identity and evaluate the species integrity of the remaining populations [4,5].

A major obstacle to these conservation efforts is the frequent natural hybridization occurring within native habitats. In its native oceanic island environments, *C. aristulifera* frequently grows in sympatry with closely related taxa, such as *C. sieboldii* and *C. discolor* [3,4]. This overlapping geographical distribution facilitates frequent natural introgression, generating complex hybrid swarms [6]. Due to frequent interspecific hybridization, phenotypic plasticity, and intermediate floral characteristics, the true genetic identities of these orchids are often masked. Consequently, relying solely on visual observation makes it exceedingly difficult to accurately identify cryptic hybrids or introgressed individuals [4]. The inability to distinguish cryptic species or introgressed individuals poses a profound risk of misidentification, which can inadvertently compromise targeted conservation strategies and misguide species restoration programs [5,7].

To overcome the diagnostic limitations of traditional morphological taxonomy and ensure the scientific validity of conservation strategies, molecular markers and genomics-based approaches have emerged as an indispensable alternative. While previous studies predominantly utilized traditional markers such as RAPD, ISSR, and AFLP to evaluate genetic relationships and identify hybrids among Korean *Calanthe* species, these markers exhibit clear limitations, lacking the ability to represent the entire genome, the high-throughput efficiency required for large-scale population screening, and base-pair level reproducibility [8]. To address these shortcomings, Genotyping-by-Sequencing (GBS), a next-generation sequencing (NGS)-based technology, has recently been actively adopted to evaluate genetic diversity and develop diagnostic markers for non-model endangered plants. As a reduced-representation genomic approach utilizing restriction enzymes, GBS provides a highly robust framework for discovering a vast number of genome-wide single nucleotide polymorphisms (SNPs) and insertion-deletion (InDel) variants with high resolution, even in Orchidaceae species with complex evolutionary histories [9,10]. Integrative approaches combining morphological phenotype data with GBS-based genomic data play a pivotal role in elucidating subtle genetic differences obscured by phenotypic plasticity and explicitly identifying cryptic hybrid individuals [4,11].

To practically apply the discovered large-scale genomic variation data to on-site conservation and mass seed propagation programs, strategic integration with Kompetitive Allele-Specific PCR (KASP) and Sequence-Characterized Amplified Region (SCAR) assays is required. The utility of GBS-derived SNP data for developing practical KASP-based molecular tools has recently been demonstrated in high-value plant species, including *Panax ginseng*, where Kim et al. [12] successfully translated genome-wide SNP markers into a cost-effective KASP genotyping platform for molecular breeding applications [12]. KASP technology provides a cost-effective platform capable of rapidly screening specific SNPs with high precision across large numbers of samples via fluorescence-based allele-specific amplification, making it well-suited for large-scale genetic purity testing and selection of endangered species [13,14]. Complementarily, SCAR markers guarantee high practicality and portability, allowing for intuitive allele diagnostics using standard PCR and electrophoresis equipment near natural habitats or restoration sites without the need for expensive fluorescence readers [15]. The combination of these two technologies establishes a validated diagnostic system that simultaneously satisfies the high-precision screening efficiency of the laboratory and the accessibility of on-site field diagnostics.

In this study, we utilized a comprehensive GBS dataset obtained from 64 *Calanthe* individuals—the same dataset reported in Kim et al. [4], re-analyzed using a more stringent marker-development filtering pipeline—to mine fixed homozygous alleles capable of clearly distinguishing pure *C. aristulifera* from hybrids. Based on these findings, we successfully developed a combination of three KASP markers (Ca-KASP1–3) and two SCAR markers (Ca-SCAR1–2). The validated KASP-SCAR molecular toolkit presented in this study will elucidate the true genetic identity of *C. aristulifera* within morphologically ambiguous *Calanthe* hybrid swarms, ultimately providing a validated molecular toolkit to safeguard the taxonomic integrity and scientifically support the restoration of endangered *C. aristulifera* populations.

## 2. Results

### 2.1. Morphological Evaluation and Sample Categorization

**Figure 1:**
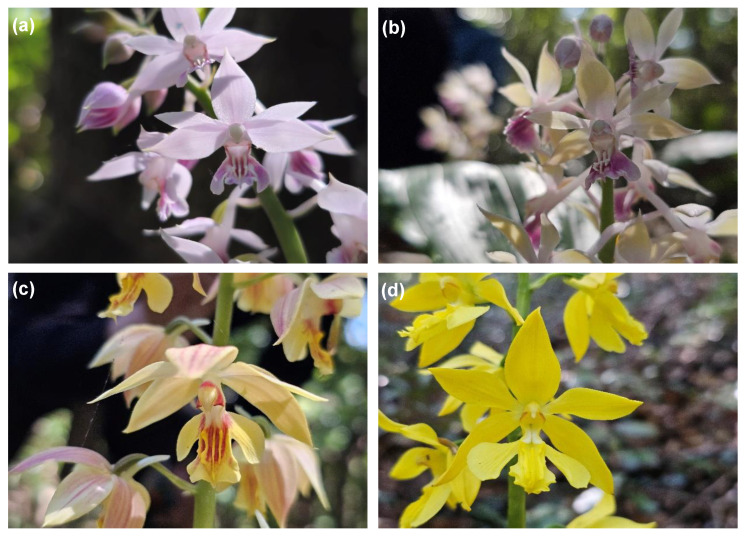
Representative floral phenotypes of the *Calanthe* taxa examined in this study. (**a**) Purebred *C. aristulifera*, exhibiting typical pale purple to pinkish petals and sepals. (**b**) *C. aristulifera* variant, displaying yellow petals and sepals. (**c**) *C. × kibanakirishima*, characterized by pale yellow petals and sepals with a reddish tint. (**d**) *C. sieboldii*, featuring bright yellow petals and sepals.

Prior to genomic analysis for molecular marker development, a total of 64 *Calanthe* individuals collected from their natural habitats were meticulously categorized based on their traditional floral phenotypes (Table 1). As illustrated in Figure 1, the collected taxa exhibited a broad and continuous spectrum of morphological variation within their sympatric habitats. (a) Purebred *C. aristulifera* typically featured pale purple to deep pinkish petals and sepals with a characteristic downward-pointing spur. In contrast, (c) the natural hybrid *C. × kibanakirishima* was characterized by pale yellow petals with a reddish tint, while (d) *C. sieboldii* displayed distinct, bright yellow floral organs.

Notably, this study identified an atypical variant (Figure 1b) that exemplified the pitfalls of morphological classification. These specific individuals (Samples 53–55) possessed the typical inflorescence and structural characteristics of *C. aristulifera*, yet expressed distinct yellow petals and sepals strongly reminiscent of *C. sieboldii*. Such an atypical color phenotype strongly suggests the presence of historical introgression or genetic instability due to incomplete morphological divergence. Therefore, to absolutely prevent any margin of error regarding the genetic stringency and diagnostic reliability of the downstream species-specific molecular markers, these variants were strictly excluded from the reference purebred pool. This frequent overlapping of visual characteristics starkly demonstrates the inherent limitations of traditional taxonomy, thereby reinforcing the fundamental rationale of this study: the critical necessity of developing precision genomics-based diagnostic tools that transcend visual evaluation.

### 2.2. GBS Data Analysis and Identification of Species-Specific Variants

**Figure 2:**
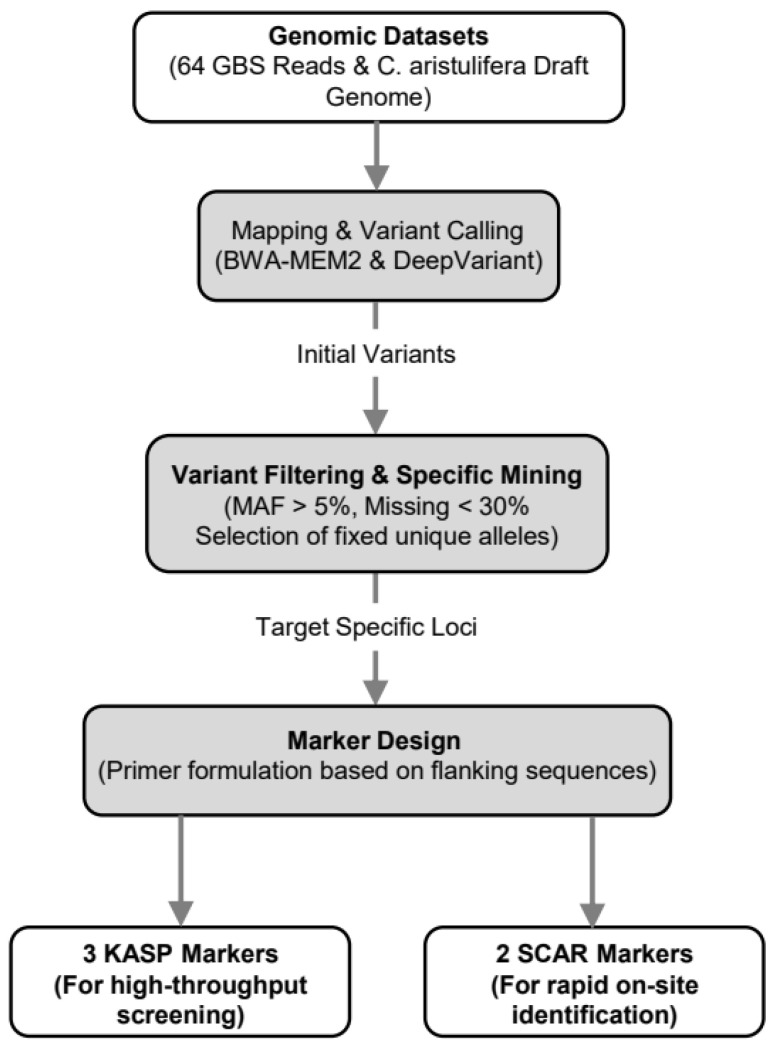
The bioinformatics pipeline for mining *C. aristulifera*-specific diagnostic markers using GBS data. A simplified flowchart illustrating the transition from genomic datasets [9] to read mapping, variant calling, stringent filtering, and the final selection of fixed homozygous loci specific to the target species for KASP and SCAR primer design.

This study utilized the GBS dataset generated and published in Kim et al. [4] (BioProject PRJNA1306762). GBS was performed using the Illumina NovaSeq X platform following the ApeKI (GCWGC) restriction enzyme digestion protocol (library fragment size 300–600 bp, major peak at 480–500 bp), generating a total of 278.8 Gbp of short reads across 64 samples (GC: 47%, Q30: 94%; average 24,802,946 raw reads per sample, 3.6 Gbp). Reads were quality-trimmed using Trimmomatic v0.39 (window size 4, leading/trailing quality ≥ 3, mean quality ≥ 15, minimum length 36 bp), aligned to the *C. aristulifera* draft reference genome using BWA-MEM2, and variants were called using DeepVariant v1.6.0 (Illumina WGS model). The average SNP coverage depth was 93.97× [4]. The draft reference genome (PacBio HiFi assembly: 5.1 million HiFi reads, 84.39 Gbp, average read length 16,546 bp, N50 16,985 bp, HiFi coverage ~7.6×; assembled genome: 9.4 Gbp, 3600 scaffolds, N50 0.49 Gbp) is publicly available under BioProject PRJNA1306762 [4]. GBS raw data for the present study are additionally deposited under BioProject PRJNA1452209 (BioSample SAMN57221933). The complete bioinformatics pipeline for marker development is illustrated in Figure 2.

For the marker development pipeline, we applied additional filters beyond the basic pipeline of Kim et al. [4] (depth ≥ 3, MAF > 5%, missing < 30%): minimum mapping quality (MAPQ) ≥ 20 and exclusion of loci deviating from Hardy–Weinberg equilibrium within *C. aristulifera* (*p* < 0.001). These additional filters reduced the variant pool from 853,301 SNPs and 55,857 InDels to 62,231 high-quality SNPs and 1271 InDels (Table 2).

From this refined dataset, 179 SNP and 107 InDel loci exhibiting fixed homozygosity exclusively within the *C. aristulifera* population were identified (Table 2). Crucially, the companion GBS population genomic study [4] provides independent molecular ground truth for evaluating marker performance. In that study, performed without reference to morphological labels, PCoA placed Sample 52 in an intermediate genetic cluster between *C. aristulifera* and *C.* sieboldii (Figure 4b in [4]). Hybrid index analysis further showed that CA1 individuals had hybrid indices of 0.93–0.99 and CA2 individuals 0.92–0.95—both consistent with pure *C. aristulifera* ancestry—while CK1 individuals showed hybrid indices of 0.48–0.50, approaching the theoretical F1 value of 0.5 (Figure 7 in [4]). Sample 52’s KASP heterozygous pattern is fully consistent with this CK1-range hybrid index, providing independent corroboration of its hybrid identity entirely independent of morphological assessment.

### 2.3. Validation and Genotyping Precision of Ca-KASP Markers

**Figure 3:**
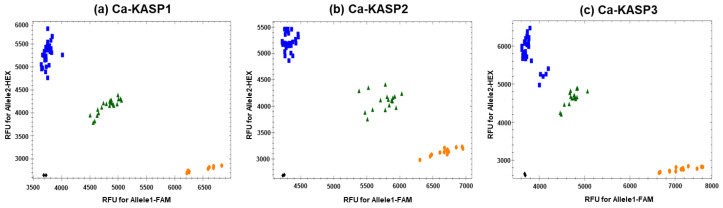
Allelic discrimination of the Ca-KASP markers. Blue squares (HEX fluorescence): *Calanthe aristulifera* purebred individuals carrying the target homozygous allele (T/T for Ca-KASP1 and 2; Deletion/Deletion for Ca-KASP3). Orange circles (FAM fluorescence): non-target *Calanthe* species carrying the alternative homozygous allele. Green triangles (FAM + HEX): heterozygous individuals indicative of hybrid genotypes. Black diamonds: negative template control (NTC). The genotypes of *Calanthe aristulifera* were clearly separated from those of other *Calanthe* species based on fluorescence signals, confirming the reliability of the Ca-KASP markers.

To support the high-throughput screening required for large-scale native populations and seed propagation programs, three KASP markers (Ca-KASP1, 2, and 3) were ultimately developed (Table 3). Allelic discrimination assays performed on all 64 individuals demonstrated clear clustering of genotypes (Figure 3). Purebred *C. aristulifera* individuals clustered exclusively along the HEX fluorescence axis (the target allele), whereas non-target related species distributed accurately along the FAM axis, and hybrids positioned themselves in the FAM + HEX (heterozygous) region.

The KASP marker set achieved a concordance of 98.4% (63/64 individuals; 95% CI: 91.6–99.7%) with morphological pre-classification (Table 4). This high concordance is a direct result of establishing a strict genetic baseline by proactively excluding the aforementioned phenotypic variants (Samples 53–55) from the reference group. The most compelling finding of this assay was the reclassification of Sample 52. This individual exhibited the perfect floral structure and color typical of *C. aristulifera*, leading to its initial classification as a purebred during morphological evaluation (Table 1). However, analysis using all three KASP markers generated a strong dual fluorescence (FAM + HEX) signal, identifying it as a morphologically cryptic hybrid. This reclassification is independently corroborated by Kim et al. [4], who reported the same individual clustered in an intermediate genetic position with a hybrid index of approximately 0.48–0.50—without any reference to morphological labels—confirming its hybrid identity through molecular evidence entirely independent of morphology. The case of Sample 52 highlights the resolving power of the KASP system in detecting subtle introgression and underscores that the implementation of molecular markers is imperative, rather than optional, in conservation biology. Formal statistical evaluation yielded a sensitivity of 100% (32/32 purebred *C. aristulifera* correctly identified as target) and specificity of 96.9% (31/32 non-target individuals correctly identified; Table 4).

### 2.4. Efficacy of Combined SCAR Markers for Rapid On-Site Identification

**Figure 4:**
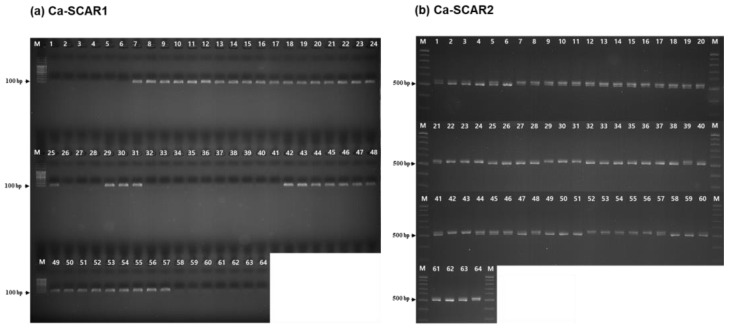
Validation of the developed SCAR markers for species identification. PCR amplification products were separated on 1.5% (Ca-SCAR1, panel (**a**) and 2.5% (Ca-SCAR2, panel (**b**) agarose gels. (**a**) Amplification results of the Ca-SCAR1 marker. Samples of *Calanthe aristulifera* showed no amplification. The amplification patterns of *C. aristulifera* were clearly separated from those of other *Calanthe* species based on the presence or absence of the 116 bp band, confirming the reliability of the Ca-SCAR1 marker. (**b**) Amplification results of the Ca-SCAR2 marker. Samples of *C. aristulifera* showed a single 516 bp band. The amplification patterns of *C. aristulifera* were clearly separated from those of other *Calanthe* species based on the band patterns, confirming the reliability of the Ca-SCAR2 marker. M: DNA ladder (100 bp for panel a; 500 bp for panel b, indicated on the left of each gel row); lanes 1–64: representative *Calanthe* individuals.

Despite the high screening efficiency of the KASP marker system, two SCAR markers (Ca-SCAR1 and 2) were additionally developed to ensure practical applicability in standard laboratories or on-site conservation areas lacking expensive fluorescence readers (Table 5). Gel electrophoresis revealed visually distinct and highly reproducible banding patterns (Figure 4). The dominant marker, Ca-SCAR1, showed a “null” allele (no amplification) exclusively for *C. aristulifera*, while consistently generating a distinct 116 bp band in non-target species. Conversely, the codominant marker, Ca-SCAR2, yielded a 516 bp band for the target species, clearly separating it from the 537 bp band of related taxa, thereby opening the possibility for heterozygote differentiation.

During the analysis, it was observed that due to the complex hybrid swarms within native habitats, incomplete lineage sorting could occasionally cause single SCAR markers to yield ambiguous results. To structurally overcome this limitation, a ‘merged diagnostic framework’ was devised, cross-validating the combined genotypic patterns of both SCAR loci (Table 6). By applying this strict criterion—combining the presence/absence of Ca-SCAR1 (O/X) with the amplicon size of Ca-SCAR2 (516/537)—the SCAR system alone achieved a concordance of 96.9% (62/64 individuals; 95% CI: 89.3–99.1%) with morphological pre-classification. Formal statistical evaluation yielded a sensitivity of 93.8% (30/32 purebred *C. aristulifera* correctly identified) and specificity of 100% (32/32 non-target individuals correctly identified; Table 6). The high concordance between the results from the laboratory-optimized KASP assays and the highly portable, cost-effective SCAR system validates the complementary reliability of the integrated molecular toolkit for identifying endangered *C. aristulifera* and realizing scientific conservation of genetic resources.

## 3. Discussion

### 3.1. Analytical Synergy: Laboratory High-Throughput vs. Field-Applicable

The strategic integration of GBS-derived SNP and InDel markers provides a high-resolution solution to the long-standing taxonomic ambiguity surrounding *Calanthe aristulifera* [1]. By successfully identifying 179 SNP and 107 InDel loci strictly specific to the target species, this research offers a higher diagnostic resolution than previous studies relying on traditional chloroplast barcodes (e.g., *matK*, *rbcL*) or nuclear ITS regions [8,16]. Such conventional markers, which are predominantly maternally inherited or subject to concerted evolution, frequently fail to capture complex bidirectional gene flow and nuclear introgression patterns, especially in recently diverged orchid lineages experiencing incomplete lineage sorting or deception-driven hybridization [16,17]. Recent comparative genomic studies, such as the assembly of the highly fragmented mitogenome of *C. sieboldii*, further underscore the extreme structural rearrangements and dynamic evolution inherent to the *Calanthe* genus [18]. In contrast, the vital transition from traditional conservation genetics to conservation genomics [19,20] has firmly established reduced-representation genomic approaches like Genotyping-by-Sequencing (GBS) as a highly robust framework for discovering genome-wide variations, unveiling subtle genetic divergence that traditional methods overlook [9,10]. The methodological framework adopted here—mining genome-wide SNPs from GBS data and converting them into cost-effective, field-deployable KASP assays—is directly analogous to recent work by Kim et al. [12] in Panax ginseng.

To translate these robust genomic findings into highly practical conservation tools—thereby bridging the persistent “conservation genetics gap” between researchers and practitioners [7]—we integrated high-throughput KASP and rapid SCAR marker systems. This dual-tier approach effectively addresses both the analytical precision and the on-site practicality required in real-world conservation genomics [21,22]. KASP technology is well-suited for the large-scale genetic auditing of ex situ living collections and germplasm banks, offering high reproducibility at the single base-pair level [13]. Recent advancements continually highlight the efficacy of KASP combined with reduced-representation sequencing in accelerating the accurate identification of endangered plant taxa [14]. Conversely, our developed SCAR system facilitates rapid, instrumentation-free identification. Specifically, the codominant Ca-SCAR2 marker produces a distinct 516 bp band that can be easily visualized via simple agarose gel electrophoresis without the need for expensive fluorescence readers [15]. It is also important to note that the successful deployment of these molecular diagnostic tools fundamentally relies on high-quality DNA isolation procedures, which remain a critical prerequisite for handling recalcitrant plant tissues [23]. The high degree of congruency between the KASP and SCAR platforms validates their complementary reliability. This integrated framework bridges the critical gap between sophisticated laboratory-based genomic research and localized on-site conservation management, enabling rapid screening to prevent the accidental introduction of non-native alleles into protected breeding programs [24,25].

A key limitation that must be transparently acknowledged is that the concordance statistics reported here (KASP 98.4%, SCAR 96.9%) are calculated against morphological pre-classification rather than a pedigree-verified reference panel. However, the morphology-blind GBS population genomic analysis of Kim et al. [4] independently corroborated the hybrid identity of Sample 52 through hybrid index analysis (0.48–0.50) and PCoA clustering, providing strong independent evidence that our marker results reflect true biological reality rather than morphological bias.

### 3.2. Overcoming Morphological Constraints and Detecting Cryptic Introgression

The paramount challenge in the conservation of *Calanthe* species is the frequent occurrence of natural hybridization among sympatric relatives, including *C. sieboldii* and *C. discolor*, within their restricted native or island-endemic habitats [3,11]. Traditional taxonomic classification, which relies heavily on floral phenotyping, is often severely confounded by complex hybrid swarms where advanced generation or backcrossed individuals seamlessly mimic the parental morphologies [6]. Our study explicitly addresses these critical limitations by categorizing a diverse phenotypic spectrum into genetically verified units, thereby exposing the unreliability of visual identification [4,5].

The most compelling evidence of this morphological constraint is demonstrated by Sample 52. Despite exhibiting a floral phenotype and structural morphology virtually indistinguishable from purebred *C. aristulifera*, all three Ca-KASP markers generated strong dual fluorescence (FAM + HEX) signals, and the combined Ca-SCAR markers also produced genotype patterns consistent with a hybrid individual. This reclassification is independently corroborated by Kim et al. [4], in which the same individual clustered in an intermediate genetic position with a hybrid index of approximately 0.48–0.50—without any reference to morphological labels. The inability to detect such “cryptic species” or masked introgressed individuals presents a profound risk to diversity conservation [5]. This phenomenon, known as “cryptic introgression,” poses a severe and often silent threat that can ultimately lead to extinction by hybridization [26]. If such morphologically cryptic hybrids are mistakenly authenticated by visual inspection and subsequently utilized as maternal plants for in vitro propagation and mass multiplication, they can precipitate outbreeding depression. This would lead to the breakdown of co-adapted gene complexes, reduced ecological fitness, and the gradual genetic erosion of the already critically endangered native populations upon reintroduction [2,25].

To systematically establish this final phenotype-genotype concordance, our analysis revealed that 59 of 64 individuals (92.2%) showed complete agreement between morphological classification and KASP genotyping. Kim et al. [4] independently confirmed that CA1 and CA2 form a single indistinguishable genetic cluster in PCoA and STRUCTURE analysis (k = 2), corroborating the identical KASP allele patterns observed for both phenotypes in the present study. The five discordant cases (Sample 52: cryptic hybrid; Samples 53–55: yellow-flowered atypical variants) clearly underscore the inherent limitations of morphological identification alone.

Furthermore, the strategic exclusion of the atypical yellow variant from our initial marker development baseline ensured the selection of diagnostic loci with maximum species specificity. Applying this rigorous “genetic filter” and the molecular framework validated here is absolutely essential for accurately identifying Evolutionarily Significant Units (ESUs) and ensuring that human-mediated reintroduction efforts do not inadvertently compromise the intrinsic genetic integrity of the wild populations [24].

### 3.3. Limitations and Future Directions

Several limitations of the present study should be acknowledged. First, as the 64-sample panel was collected exclusively from Hongdo Natural Reserve, Korea [4], applicability of these markers to *C. aristulifera* populations in Japan and China requires independent validation. Second, incomplete lineage sorting cannot be excluded given the relatively recent divergence of *C. aristulifera* from sympatric congeners, which may occasionally generate false-positive or false-negative results. Third, advanced-generation backcross hybrids that have largely recovered the *C. aristulifera* genotype could theoretically evade detection. Fourth, no formal sensitivity analysis of the fixation threshold was conducted. Fifth, the concordance metrics reported here are calculated against morphological pre-classification; true diagnostic accuracy in the strict statistical sense requires a validated reference panel with known pedigree, which we recommend as a priority for future studies.

### 3.4. Evolutionary Significance and Conservation Policy Implications

From an evolutionary perspective, the successful identification of fixed homozygous alleles uniquely specific to *C. aristulifera* suggests that these genomic loci are likely associated with critical reproductive isolation barriers or reside within genomic regions under strong purifying selection [1,27]. The effective legal protection and sustainable management of critically endangered orchids require a robust, evidence-based scientific consensus on species boundaries. The dual-marker system established here provides high diagnostic precision, allowing for the reliable detection of F1 hybrids as well as complex backcrosses, which have historically been indistinguishable during standard field ecological surveys.

The implementation of our molecular diagnostic framework empowers conservation authorities and restoration centers to establish definitive “Genetic Safety Zones” by identifying and mapping purebred populations with high confidence. In the context of the accelerating global decline in Orchidaceae biodiversity, population genomics provides high precision for wildlife conservation, making rapid, cost-effective, and accurate genetic authentication a fundamental prerequisite prior to any conservation intervention [22,28]. This ensures that seeds collected for ex situ seed banking or tissue culture are genetically pure. Ultimately, this study demonstrates that genomics-informed diagnostic tools are not merely supplementary enhancements, but are absolutely indispensable for the long-term survival, rigorous genetic monitoring, and successful habitat restoration of endangered *C. aristulifera* populations currently facing complex anthropogenic threats and evolutionary pressures [8,25].

## 4. Materials and Methods

### 4.1. Plant Materials and DNA Extraction

A total of 64 *Calanthe* individuals, encompassing purebred *C. aristulifera*, its morphological variants, related sympatric species, and natural hybrids, were utilized in this study (Table 1). The representative floral phenotypes of these taxa are illustrated in Figure 1. Fresh leaf tissues were collected from their natural habitats on the oceanic island and rapidly frozen in liquid nitrogen. Total genomic DNA was extracted using the modified CTAB (cetyltrimethylammonium bromide) method [23]. The exact DNA concentration of each sample was accurately quantified using a Qubit 4 Fluorometer (Thermo Fisher Scientific, Waltham, MA, USA), yielding DNA concentrations ranging from 20 to 112 ng/µL, which ensured optimal starting material for downstream genomic analyses.

Although Samples 53–55 cluster genetically with typical *C. aristulifera* individuals in GBS-based PCoA and STRUCTURE analyses [4], their atypical yellow floral pigmentation resembling *C. sieboldii* led us to conservatively exclude them from the purebred reference pool to maximize phenotype-independent marker specificity.

### 4.2. GBS Data Analysis and Locus Selection

This study utilized the GBS dataset generated and published in Kim et al. [4]. GBS was performed using the ApeKI (GCWGC) enzyme digestion protocol (library fragment size 300–600 bp, major peak 480–500 bp) and Illumina NovaSeq X paired-end sequencing (2 × 151 bp), generating 278.8 Gbp across 64 samples (GC: 47%, Q30: 94%; avg. 24,802,946 reads/sample, 3.6 Gbp total). The *C. aristulifera* draft reference genome (PacBio HiFi assembly; 5.1 million HiFi reads, 84.39 Gbp, avg. read length 16,546 bp, N50 16,985 bp, coverage ~7.6×; assembled genome 9.4 Gbp, 3600 scaffolds, N50 0.49 Gbp) is publicly available under BioProject PRJNA1306762 [4]. GBS raw data are additionally deposited under BioProject PRJNA1452209 (BioSample SAMN57221933) for the present study.

Reads were quality-trimmed using Trimmomatic v0.39 (window size 4, leading/trailing quality ≥ 3, mean quality ≥ 15, minimum read length 36 bp) and aligned to the draft genome using BWA-MEM2 [4]. Variants were called using DeepVariant v1.6.0 (Illumina WGS model; avg. SNP depth 93.97×). For the marker development pipeline of this study, the following additional filters were applied beyond the basic pipeline of Kim et al. [4] (depth ≥ 3, MAF > 5%, missing < 30%): minimum mapping quality (MAPQ) ≥ 20 and exclusion of loci deviating from Hardy–Weinberg equilibrium within *C. aristulifera* (*p* < 0.001). These filters reduced the variant pool from 853,301 SNPs and 55,857 InDels to 62,231 high-quality SNPs and 1271 InDels (Table 2).

The allele mining procedure proceeded in two stages consistent with the sample composition of Kim et al. [4]. In Stage 1, all 36 morphologically classified *C. aristulifera* individuals (typical form: n = 33; atypical yellow variant: n = 3) were included. In Stage 2, after KASP validation identified Sample 52 as a cryptic hybrid and Samples 53–55 were excluded as phenotypic variants, the confirmed purebred training set was reduced to n = 32. A ‘fixed homozygous allele’ was operationally defined as a locus at which (1) 100% of the 32 confirmed purebred individuals carried the same homozygous allele, and (2) this allele was completely absent (frequency = 0%) in all non-target individuals. This definition is consistent with the species-specific SNP Group 2 (540 SNPs) identified through heatmap clustering in Kim et al. [4].

### 4.3. KASP Marker Design and Genotyping

Based on the selected robust SNP and InDel loci, three Kompetitive Allele-Specific PCR (KASP) markers (Ca-KASP1, Ca-KASP2, and Ca-KASP3) were designed. Primers were designed using Primer3 (v2.6.0) with a target Tm of 58–62 °C, GC content of 40–60%, and primer length of 20–30 nt. For each marker, two allele-specific forward primers tailed with universal FAM or HEX fluorescent sequences from LGC Biosearch Technologies (Teddington, UK) and one common reverse primer were synthesized [13]. The KASP assays were performed in a PCR mixture containing 4 ng of genomic DNA and the KASP-TF V4.0 2X Master Mix/Standard ROX (LGC Genomics, Teddington, UK). PCR amplification and endpoint fluorescence reading were conducted utilizing a CFX96™ Real-Time PCR Detection System (Bio-Rad Laboratories, Hercules, CA, USA). The thermal cycling conditions included an initial activation step at 94 °C for 15 min, followed by 10 touchdown cycles (94 °C for 20 s; annealing/extension starting at 61 °C and decreasing by 0.6 °C per cycle to 55 °C for 60 s), and 26 standard cycles (94 °C for 20 s; 55 °C for 60 s). End-point fluorescence signals (FAM and HEX) were clustered and visualized using the CFX Maestro Software v1.1 (Bio-Rad Laboratories, Hercules, CA, USA).

### 4.4. SCAR Marker Development and PCR Amplification

To develop a rapid and field-applicable diagnostic system, two Sequence-Characterized Amplified Region (SCAR) markers (Ca-SCAR1 and Ca-SCAR2) were designed targeting specific large InDel regions identified from the GBS data. Primers were designed to span the InDel region with expected amplicon size differences of ≥20 bp between alleles for unambiguous gel discrimination. Polymerase chain reaction (PCR) amplification was conducted using TOPsimple™ PCR PreMIX-HOT (Enzynomics, Daejeon, Republic of Korea). The reaction mixture comprised 4 ng of genomic DNA template, the 2× PreMIX, and 10 pmol/µL each of the forward and reverse primers. The PCR thermal profile consisted of an initial denaturation at 95 °C for 5 min, followed by 35 cycles of denaturation at 95 °C for 30 s, a standardized annealing step at 63 °C for 30 s, and extension at 72 °C for 45 s, with a final extension at 72 °C for 5 min.

For visualization, 5 µL of the amplified SCAR products were separated by electrophoresis at 210 V under marker-specific optimized conditions: Ca-SCAR1 products were resolved on a 1.5% agarose gel for 18 min, whereas Ca-SCAR2 products were separated on a 2.5% agarose gel for 100 min. The gels were stained with ethidium bromide (EtBr) and visualized under an ultraviolet (UV) transilluminator to determine the presence and exact size of the species-specific bands [15].

## 5. Conclusions

In conclusion, this study successfully established a robust molecular diagnostic framework for the critically endangered *Calanthe aristulifera* by leveraging high-throughput GBS data. We developed highly accurate KASP (98.4%) and SCAR (96.9%) markers based on 179 SNP and 107 InDel loci strictly specific to the target species. This dual-marker system effectively overcomes the inherent limitations of traditional morphological identification, as demonstrated by the precise detection of cryptic introgression in morphologically ambiguous individuals. The strategic integration of high-throughput laboratory assays and rapid, instrumentation-free field screening provides a versatile and cost-effective toolkit for conservation practitioners. Ultimately, the implementation of these genomics-informed markers will be instrumental in authenticating purebred populations, preventing outbreeding depression, and guiding the strict legal protection and scientific restoration of endangered *Calanthe* species.

## Figures and Tables

**Table 1:** Summary of the *Calanthe* accessions used in this study, comparing morphological phenotypes with the newly validated genetic identification.

Morphological ID	Floral Phenotype (Description)	No. of Samples	Genetic ID (This Study)
*C. aristulifera*	Pale purple to pinkish petals and sepals (Typical form)	33	*C. aristulifera* (Target purebred)
*C. aristulifera* (Variant)	Yellow petals and sepals (Atypical variant)	3	Non-target (Variant excluded)
*C. sieboldii*	Bright yellow petals and sepals	16	Non-target (*C. sieboldii/*Excluded)
C. × *kibanakirishima*	Pale yellow with reddish tint	12	Non-target (Hybrid)

**Table 2:** Summary of variant discovery and filtering for identifying *C. aristulifera*-specific loci.

Variant Type	Initial Variants Identified	High-Quality Variants (Filtered) *	*C. aristulifera*-Specific Loci **
SNP	853,301	62,231	179
InDel	55,857	1271	107 ***

* Filtered at depth ≥ 3 [4], MAF > 5%, missing data < 30%, plus additional filters MAPQ ≥ 20 and HWE *p* > 0.001 (this study). ** Target species population is entirely homozygous for a unique allele not present in other taxa. *** The same InDel locus was used for both KASP (Ca-KASP3) and SCAR (Ca-SCAR1) marker design.

**Table 3:** Primer sequences and target genomic loci characteristics of the developed KASP markers.

Marker Name	Variation Type	Target Genomic Position	Primer Type	Primer Sequence (5′–3′)
Ca-KASP1	SNP	ptg0118431_46155	FAM	TCCACTTCAAGAACCACCTCCGCTCC
			HEX	TCCACTTCAAGAACCACCTCCGCTCT
			Common	AGGGACTCACTAAGCTGATTACGGATGC
Ca-KASP2	SNP	ptg0143131_40291	FAM	AGTTTGGCATCCAAGAGAGTCCTTAGACGC
			HEX	AGTTTGGCATCCAAGAGAGTCCTTAGACGT
			Common	GCACTATTTGTGGTCCTACATGCTTTCTGA
Ca-KASP3	InDel	ptg0002201_460974	FAM	CATGAGTGCAAATTGACCTCAAGGAAA
			HEX	GCATGAGTGCAAATTGACCTCAACATA
			Common	GCCATATTTAGTCATGTGAGGGGTTGTT

**Table 4:** Diagnostic performance of the developed Ca-KASP markers for *C. aristulifera* identification.

Marker Name	Target Genotype (*C. aristulifera*)	Non-Target Genotype	Heterozygote	Concordance (n = 64)	Sensitivity/Specificity (95% CI)
Ca-KASP1	T/T (HEX)	C/C (FAM)	C/T (FAM + HEX)	98.4% (63/64)	100%/96.9% (91.6–99.7%) *
Ca-KASP2	T/T (HEX)	C/C (FAM)	C/T (FAM + HEX)	98.4% (63/64)	100%/96.9% (91.6–99.7%) *
Ca-KASP3	Del/Del (HEX)	Ins/Ins (FAM)	Ins/Del (FAM + HEX)	98.4% (63/64)	100%/96.9% (91.6–99.7%) *

* Concordance with morphological pre-classification; 95% CI by Wilson score method (n = 64). Sensitivity = 100% (32/32 purebreds identified). Specificity = 96.9% (31/32 non-targets identified). The single discordant case (Sample 52) was independently confirmed as a hybrid by GBS-based hybrid index analysis (0.48–0.50) in Kim et al. [4].

**Table 5:** Primer sequences and expected amplicon sizes of the developed SCAR markers.

Marker Name	Primer Type	Target Genomic Position	Primer Sequence (5′–3′)	Expected Size (bp)
Ca-SCAR1	Dominant	ptg0002201_460974	F-CATGAGTGCAAATTGACCTCAAGGAAA	None/116
			R-GCCATATTTAGTCATGTGAGGGGTTGTT	
Ca-SCAR2	Codominant	ptg0102101_248793	F-AGCATGAGAGGGCGCAAAGG	516/537
			R-AACGCATCCCCAATCCCTGC	(or 516/537)

**Table 6:** Diagnostic performance of combined Ca-SCAR markers for *C. aristulifera* identification.

Species	Ca-SCAR1	Ca-SCAR2	Combined Pattern	Concordance	Sensitivity/Specificity (95% CI)
*C. aristulifera* (Target)	Absent (X) or Present (O)	516 or 516/537 bp	X:516, X:516/537, O:516	96.9% (62/64) *	93.8%/100% (89.3–99.1%) **
Other *Calanthe* spp.	Present (O)	537 or 516/537 bp	O:537, O:516/537	N/A	N/A

* Concordance with morphological pre-classification; 95% CI by Wilson score method (n = 64). ** Sensitivity = 93.8% (30/32 purebred *C. aristulifera* correctly identified). Specificity = 100% (32/32 non-targets correctly identified). Two discordant samples (Samples 48 and 52).

## Data Availability

The raw genotyping-by-sequencing data presented in this study are openly available in the NCBI Sequence Read Archive (SRA) at [https://www.ncbi.nlm.nih.gov/sra], (accessed on 10 April 2026), BioProject ID PRJNA1452209. Other relevant data are contained within the article and its Supplementary Materials.

## Supplementary Materials

The following supporting information can be downloaded at: https://www.mdpi.com/article/10.3390/plants15101453/s1, Table S1: Comprehensive sample information, DNA quantification, and genotyping results for 64 *Calanthe* individuals. Table S2: Comprehensive list of primer sequences and PCR conditions for all Ca-KASP and Ca-SCAR markers developed in this study.
